# Combined transplantation of mesenchymal stem cells and endothelial progenitor cells for tissue engineering: a systematic review and meta-analysis

**DOI:** 10.1186/s13287-016-0390-4

**Published:** 2016-10-10

**Authors:** Kunming Sun, Zheng Zhou, Xinxin Ju, Yang Zhou, Jiaojiao Lan, Dongdong Chen, Hongzhi Chen, Manli Liu, Lijuan Pang

**Affiliations:** 1 Department of Pathology and Key Laboratory for Xinjiang Endemic and Ethnic Diseases (Ministry of Education), Shihezi University School of Medicine, North 2nd Road, Shihezi, 832002 Xinjiang China; 2 Department of Pathology, First Affiliated Hospital to Shihezi University School of Medicine, Shihezi, China; 3 Department of Stomatology, First Affiliated Hospital to Shihezi University School of Medicine, Shihezi, China

**Keywords:** Mesenchymal stem cells, Endothelial progenitor cells, Angiogenesis, Tissue engineering, Cell transplantation, Systematic review, Meta-analysis

## Abstract

**Background:**

Combined cell implantation has been widely applied in tissue engineering in recent years. In this meta-analysis, we aimed to establish whether the combined transplantation of mesenchymal stem cells (MSCs) and endothelial progenitor cells (EPCs) promotes angiogenesis and tissue repair, compared with transplantation of a single cell type, following tissue injury or during tissue regeneration.

**Methods:**

The electronic databases PubMed, EMBASE, MEDLINE, Chinese Biomedical Literature, and China National Knowledge Infrastructure were searched in this systematic review and meta-analysis. Eighteen controlled preclinical studies involving MSC and EPC transplantation in animal models of disease, or in coculture in vitro, were included in this review. The vessel density and other functional indexes, which were classified according to the organ source, were used to evaluate the efficiency of cotransplantation. Publication bias was assessed.

**Results:**

There was no obvious difference in angiogenesis following combined cell transplantation (EPCs and MSCs) and transplantation of EPCs alone; however, an improvement in the function of damaged organs was observed following cotransplantation. In addition, combined cell transplantation significantly promoted tissue recovery in cardiovascular disease, cerebrovascular disease, and during bone regeneration. Compared with combined transplantation (EPCs and MSCs) and transplantation of MSCs alone, cotransplantation significantly promoted angiogenesis and bone regeneration, as well as vessel revascularization and tissue repair in cerebrovascular disease; however, no obvious effects on cardiovascular disease were observed.

**Conclusions:**

As an exploratory field in the discipline of tissue engineering, MSC and EPC cotransplantation offers advantages, although it is essential to assess the feasibility of this approach before clinical trials can be performed.

## Background

Following injury, the repair and regeneration of tissue requires the availability of a sufficient blood supply [1]. Numerous studies have demonstrated that angiogenesis is an important mechanism underlying the repair and functional recovery of injured tissue [2]. Therefore, it is necessary to develop approaches that promote angiogenesis and neovascularization, enabling the repair and regeneration of damaged tissue and dysfunctional cells. In recent years, several reports have described the application of cell-based therapies, involving the transplantation of a combination of cell types in regenerative medicine. Mesenchymal stem cells (MSCs) are a type of adult stem cells derived from the bone marrow with multidirectional differentiation potential, which differentiate into various cell types, according to their specific microenvironment, and also participate in the regeneration of blood vessels and damaged tissues [3]. In contrast, endothelial progenitor cells (EPCs) are mononuclear cells that circulate in the blood and are derived from different tissues. They can also participate in vascular repair by migrating to distant vessels, differentiating into mature endothelial cells (ECs), and replacing old and injured ECs [4]. It is expected that transplantation of a combination of both types of cells should compensate for the limitations of transplantation of either EPCs or MSCs alone, because the former are unable to differentiate into cardiocytes in vivo [11], whereas the latter preferentially differentiate into tissue cells but fail to elicit an improvement in tissue function owing to a lack of specificity [5]. Accordingly, cell therapy using a combination of both cell types has been performed in various studies with the aim of achieving synergistic effects in terms of angiogenesis and tissue regeneration. The present systematic review and meta-analysis was conducted to assess the effectiveness of cotransplantation on the repair of damaged tissue and to provide novel insights into the potential utility of such therapies in regenerative medicine.

## Methods

### Search strategy

A systematic search of relevant articles was performed in accordance with the recommendations of the Preferred Reporting Items for Systematic Reviews and Meta-Analyses (PRISMA) guidelines [6]. The following terms were used as keywords when searching the electronic databases PubMed, EMBASE, MEDLINE, Chinese Biomedical Literature (CBM), and China National Knowledge Infrastructure: (MSCs OR mesenchymal stem cells OR mesenchymal stromal cells) AND (EPCs or endothelial progenitor cells).

### Eligibility criteria

The eligibility criteria for including articles in this systematic review and meta-analysis were as follows: (1) all studies included were controlled comparison studies involving stem/progenitor cells and coimplantation in animals in vivo or coculturing of cells isolated from animals in vitro; (2) all studies included had at least two groups, an experimental group (EPCs and MSCs, coimplantation/coculture) and/or a control group (MSCs or EPCs alone); and (3) English or Chinese published papers and theses were included.

### Exclusion criteria

The exclusion criteria for including articles in this systematic review and meta-analysis were reviews and articles that: (1) were duplicated previously; (2) had insufficient statistical data; or (3) had a lack of control groups.

### Publication bias

Using vessel density as the main parameter presented publication bias.

### Data extraction

All studies were read and all data were extracted independently by two reviewers (KMS and ZZ). Disagreements were resolved by a third reviewer (LJP). Data were extracted following a standard format: the first author’s name, year of publication, animal species, numbers in the intervention and control groups, cell numbers in both groups, cell-injection time after generating the animal model, measurement time for the indices, and other parameters measured in the studies that were also included in this meta-analysis. In addition to vessel density, left ventricular systolic pressure (LVSP), left ventricular end-diastolic pressure (LVEDP), the rate of increase in the maximum left ventricular pressure (+d*p*/d*t*), and the rate of decrease in the maximum left ventricular pressure (−d*p*/d*t*) were chosen as functional indices for evaluating the effect on left ventricular function via echocardiography [7]. The activity of alkaline phosphatase (ALP) in cells was used as an early marker of osteogenic differentiation [8]. Brain-derived neurotrophic factor (BDNF) exerts protective effects against ischemia and hypoxia-induced brain injury [9]. All parameters were dependent on their organ sources to estimate the efficacy of cell therapies [9, 10].

### Data analysis

All data were analyzed by presenting pooled relative risks and 95 % confidence intervals using Review Manager Version 5.3. Because heterogeneity was reported in terms of vessel density, LVSP, LVEDP, +d*p*/d*t*, and –d*p*/d*t*, a random-effects model was used, and heterogeneity was evaluated using the *I* ^2^ statistic. In the absence of sufficient data for pooling, the results of individual studies are presented descriptively. Sensitivity analysis could not be performed because the data were complex. We extracted key parameters that may affect end therapy, such as the animal species used, the type and number of injected cells, the time of cell injection after modeling, and the time of measurement following the injection of cells. Small-study effects were explored using funnel plots.

## Results

### Search results and study characteristics

**Fig. 1:**
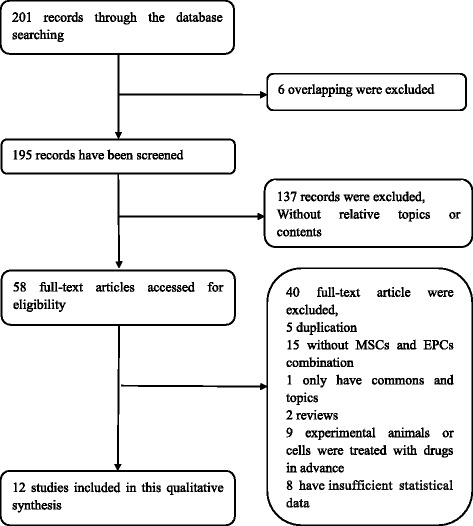
Flow diagram showing included and excluded studies

Of the 201 articles that were identified by our search, only 18 fulfilled the eligibility criteria of this study (Fig. 1). These 18 articles were all controlled comparison studies reporting in-vivo and/or in-vitro studies of cardiovascular disease (myocardial infarction, six studies) [2, 3, 11–14], cerebrovascular disease (cerebral ischemic injury, three studies) [9, 10, 15], bone-related disease [16], or bone regeneration (femoral head necrosis, eight studies) [8, 17–23].

## Meta-analysis

### The main parameter: vessel density

**Fig. 2:**
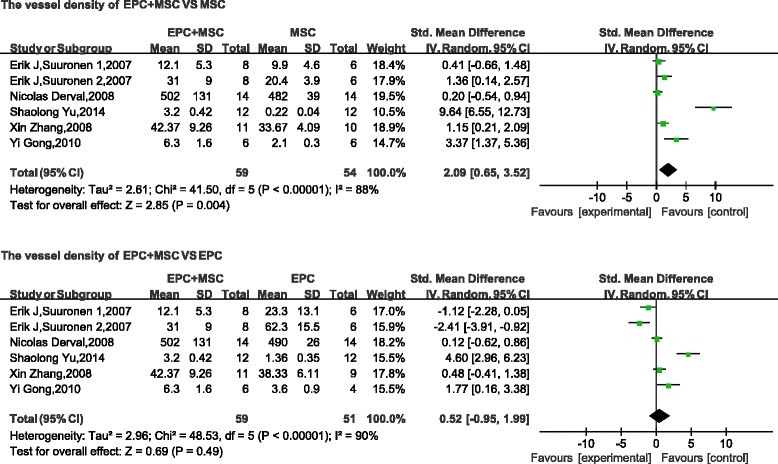
Meta-analyses of parameters. Comparison of vessel density of the combined-transplantation group versus that of the single-transplantation group. *CI* confidence interval, *EPC* endothelial progenitor cell, *IV* independent variable, *MSC* mesenchymal stem cell, *SD* standard deviation

**Fig. 3:**
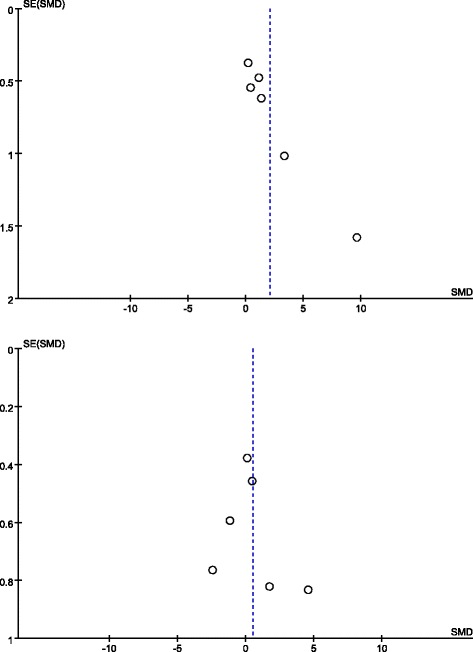
Funnel plot of vessel density. *Dotted line* shows the overall estimated standard mean difference. No obvious evidence for publication bias was found. *SE* standard error, *SMD* standard mean difference, *EPC* endothelial progenitor cell, *MSC* mesenchymal stem cell

The supply of blood to the site of injury is important for tissue regeneration, and vascular density is a measure of local tissue angiogenesis and tissue repair. Therefore, most studies have reported the use of blood vessel markers to measure vessel densities by immunohistochemistry. The vessel densities of combined-transplantation and single-transplantation groups were analyzed as continuous variables, using the mean and standard deviation. A pooled analysis of five in-vivo studies showed that the vessel density was 2.09 times higher in the combined groups than in the MSC group (standard mean difference, 2.09; 95 % CI, 0.65–3.52; *p* < 0.05; Fig. 2). Compared with the EPC group, the results of the combined-transplantation group were not significantly different (standard mean difference, 0.52; 95 % CI, −0.95 to 1.99; *p* > 0.1; Fig. 2). The funnel plot of the vessel density of the cotransplantation and single-transplantation groups revealed that their values were distributed around the overall estimate, with no obvious publication bias (Fig. 3).

### Cardiovascular diseases

**Fig. 4:**
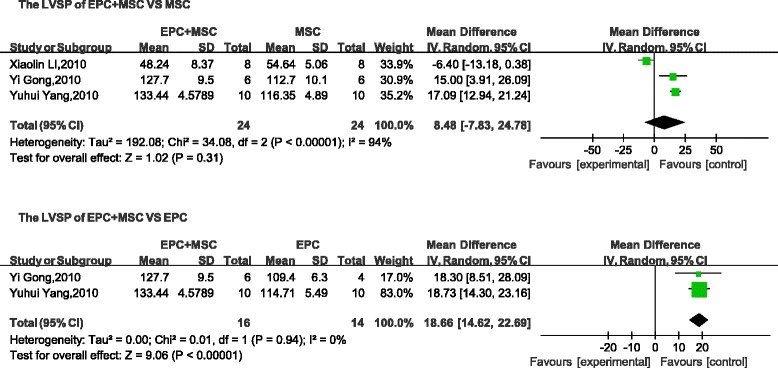
Meta-analyses of parameters. Comparison of cardiovascular function of the combined-transplantation group versus that of the single-transplantation group: LVSP. *CI* confidence interval, *EPC* endothelial progenitor cell, *IV* independent variable, *MSC* mesenchymal stem cell, *SD* standard deviation

**Fig. 5:**
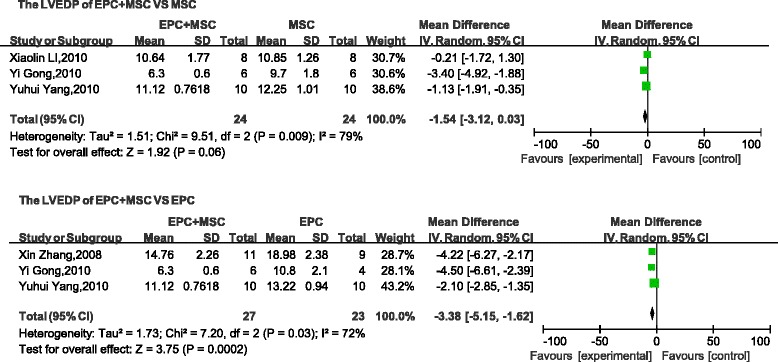
Meta-analyses of parameters. Comparison of cardiovascular function of the combined-transplantation group versus that of the single-transplantation group: LVEDP. *CI* confidence interval, *EPC* endothelial progenitor cell, *IV* independent variable, *MSC* mesenchymal stem cell, *SD* standard deviation

**Fig. 6:**
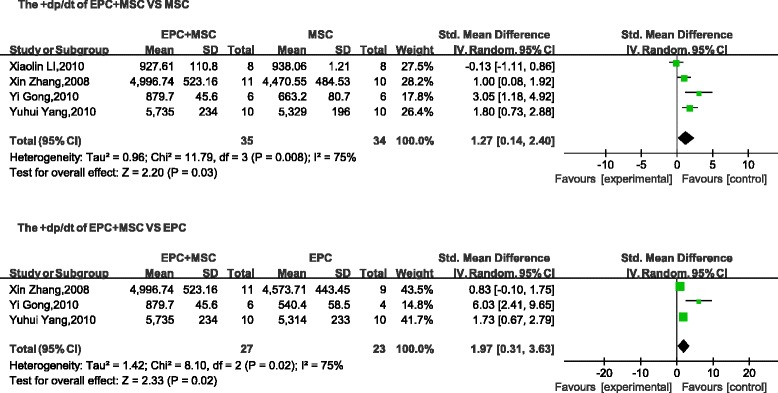
Meta-analyses of parameters. Comparison of cardiovascular function of the combined-transplantation group versus that of the single-transplantation group: +d*p*/d*t. CI* confidence interval, *EPC* endothelial progenitor cell, *IV* independent variable, *MSC* mesenchymal stem cell, *SD* standard deviation

**Fig. 7:**
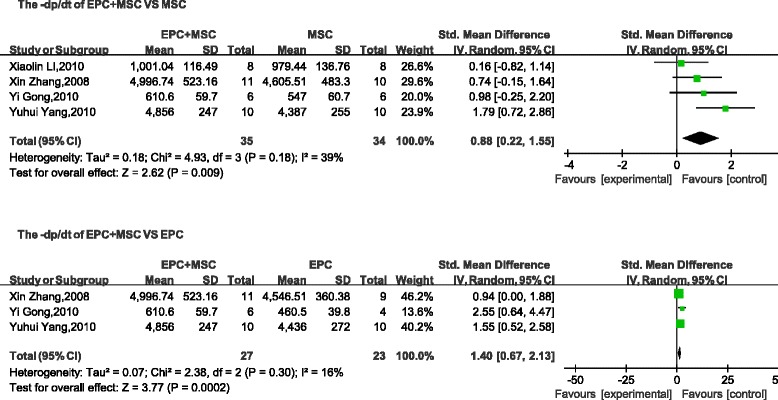
Meta-analyses of parameters. Comparison of cardiovascular function of the combined-transplantation group versus that of the single-transplantation group: –d*p*/d*t. CI* confidence interval, *EPC* endothelial progenitor cell, *IV* independent variable, *MSC* mesenchymal stem cell, *SD* standard deviation

Four parameters (LVSP, LVEDP, +d*p*/d*t*, and − d*p*/d*t*) used to describe cardiac function were selected as evaluation indices. No differences were found between the LVSP (mean difference, 8.48; 95 % CI, −7.83 to 24.78; *p* > 0.1; Fig. 4) and the LVEDP (mean difference, −1.54; 95 % CI, −3.12 to 0.03; *p* > 0.05; Fig. 5) of the combined groups and the control (MSC transplantation) group. However, compared with the + d*p*/d*t* and − d*p*/d*t* values obtained with MSC transplantation, the cotransplantation group was significantly different (+d*p*/d*t*: mean difference, 1.27; 95 % CI, 0.14–2.40; *p* < 0.05; Fig. 6; −d*p*/d*t*: mean difference, 0.88; 95 % CI, 0.22–1.55; *p* < 0.05; Fig. 7). A comparison between the combined transplantation group and the EPC-alone transplantation group revealed that the former exhibited a higher LVSP (mean difference, 18.66; 95 % CI, 14.62–22.69; *p* < 0.05; Fig. 4), +d*p*/d*t* (standard mean difference, 1.97; 95 % CI, 0.31–3.63; *p* < 0.05; Fig. 6), and − d*p*/d*t* (standard mean difference, 1.40; 95 % CI, 0.67–2.13; *p* < 0.05; Fig. 7), but a lower LVEDP (mean difference, −3.38; 95 % CI, −5.15 to −1.62; *p* < 0.05; Fig. 5).

### Femoral head necrosis and bone regeneration

**Fig. 8:**
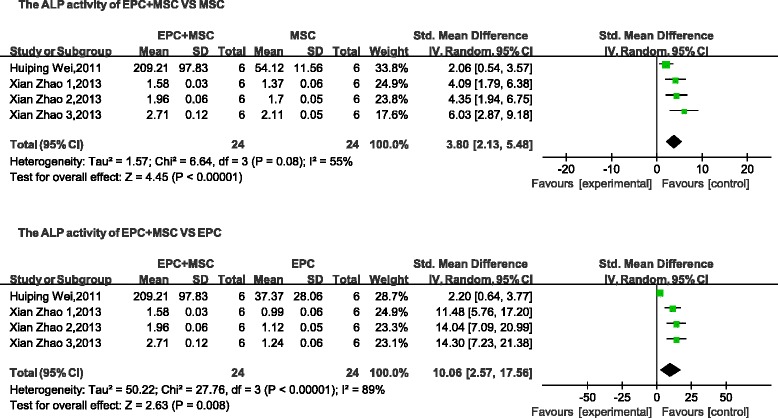
Comparison of ALP of the combined-transplantation group versus that of the single-transplantation group. *CI* confidence interval, *EPC* endothelial progenitor cell, *IV* independent variable, *MSC* mesenchymal stem cell, *SD* standard deviation

The nine studies related to bone disease or bone regeneration [8, 16–23] provided evidence that cotransplantation or coculture with both cell types improved osteogenic ability and facilitated bone repair and regeneration in comparison with those values measured after transplanting MSCs or EPCs alone. Five of these studies (5/9) reported the detection of ALP activity in coculture of heterogeneous cell types in vitro. Meta-analysis of pooled data (2/5) revealed that, in the combined-transplantation (MSCs and EPCs) groups, ALP activity in cultured cells was significantly higher than observed in cells of the MSC-alone group (standard mean difference: 3.80; 95 % CI, 2.13–5.48; *p* < 0.05) or in the EPC-alone group (standard mean difference: 10.06; 95 % CI, 2.57–17.56; *p* < 0.05, Fig. 8). According to one of the included studies (1/9), the coculture groups exhibited higher osteogenic ability at BMSC:EPC ratios of 2:1 and 1:1 [16]. In four studies (4/9), it was reported that when both cocultured cell types were seeded on β-tricalcium phosphate, partially deproteinized biologic bone, or similar biomaterials, bone formation in the combined-transplantation group was enhanced relative to the other control groups (MSCs or EPCs alone), and they also represented a potential osteogenic construct for in-vivo applications [8, 17, 18, 20]. Five of nine studies utilizing immunohistochemistry in vivo reported that coimplantation not only enhanced the bone height and bone volume, but also increased the blood vessel density and facilitated revascularization of bone tissue [8, 17, 20, 22, 23].

### Cerebrovascular disease

**Fig. 9:**
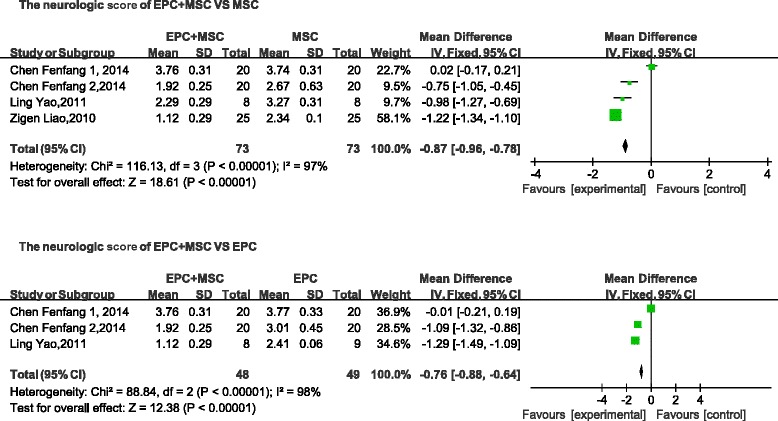
Comparison of cerebrovascular function of the combined-transplantation group versus that of the single-transplantation group: neurological impairment score. *CI* confidence interval, *EPC* endothelial progenitor cell, *IV* independent variable, *MSC* mesenchymal stem cell, *SD* standard deviation

**Fig. 10:**
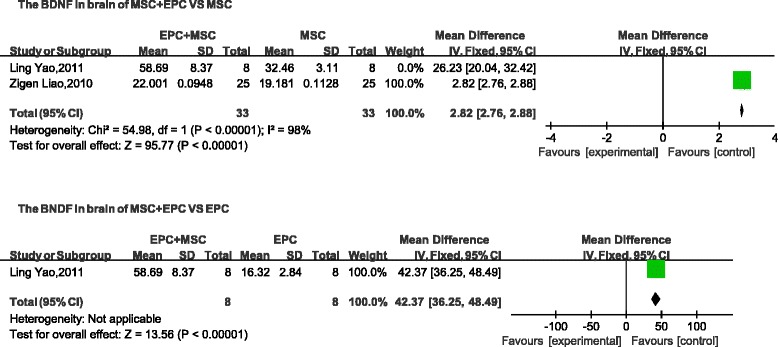
Comparison of cerebrovascular function of the combined-transplantation group versus that of the single-transplantation group: BDNF. *CI* confidence interval, *EPC* endothelial progenitor cell, *IV* independent variable, *MSC* mesenchymal stem cell, *SD* standard deviation

**Fig. 11:**
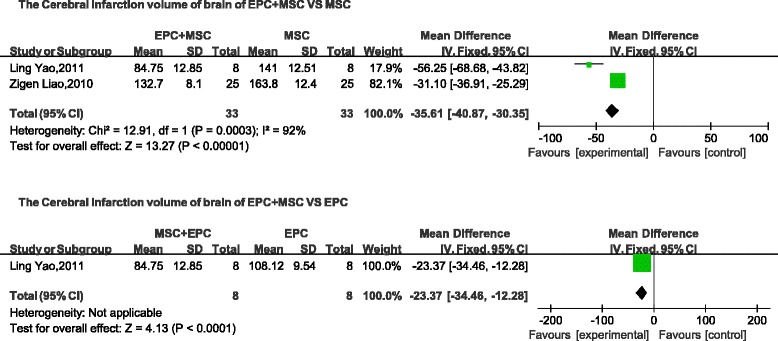
Comparison of cerebrovascular function of the combined-transplantation group versus that of the single-transplantation group: cerebral infarction volume of the brain. *CI* confidence interval, *EPC* endothelial progenitor cell, *IV* independent variable, *MSC* mesenchymal stem cell, *SD* standard deviation

The following three indices were used to describe nerve function: neurological impairment score, BDNF, and cerebral infarction volume. The neurological impairment score and the cerebral infarction volume are negatively correlated with tissue repair. Neurological impairment was lower in the combined-transplantation group than in the MSC-alone group (mean difference, −0.87; 95 % CI, −0.96 to −0.78, *p* < 0.01; Fig. 9). The levels of BDNF in the brain were higher in the combined-transplantation group than in the MSC-alone group (mean difference, 2.82; 95 % CI, 2.76–2.88, *p* < 0.01; Fig. 10). The cerebral infarction volume was lower in the cotransplantation group than in the MSC-alone group (mean difference, −35.61; 95 % CI, −40.87 to −30.35; *p* < 0.01; Fig. 11). In addition, the neurological impairment score of the combined-transplantation group was lower than that of the EPC-alone group (mean difference, −0.76; 95 % CI, −0.88 to −0.64, *p* < 0.01; Fig. 9). The levels of BDNF in the brain were higher in the combined-transplantation group than in the EPC-alone group (mean difference, 42.37; 95 % CI, 36.25–48.49, *p*  < 0.01; Fig. 10). The cerebral infarction volume of the brain in the combined transplantation group was lower than in the EPC-alone group (mean difference, -23.37; 95 % CI, −34.46 to −12.28, *p* < 0.01; Fig. 11).

### Sensitivity analyses

Sensitivity analyses could not be conducted because of the small amount of available data. Related data are presented in Table 1.

**Table 1:** Characteristics of the included studies

First author, year	Animal	Number of animals used for intervention, EPC + MSC	Number of animals used for controls, EPC/MSC	Number of cells used for intervention, EPC + MSC	Number of cells used as controls, EPC/MSC	Time of cell therapy after making the model	Time point of index measure	Index of measure
Cardiovascular								
Suuronen, 2007 [2]	Rats	8	7/6	5 × 10^5^ + 5 × 10^5^	1 × 10^6^/1 × 10^6^	21 days	28 days	1. LVEF, FS
								2. Infarct area
								3. Infarct length
								4. Heart rate
								5. Arteriole density
Derval, 2008 [3]	Mice	14	14/14	5 × 10^5^ + 5 × 10^6^	5 × 10^5^/5 × 10^6^	30 days	45 days	1. Capillary density
								2. Scar thickness
								3. Infarct size
								4. Heart rate
								5. d*p*/d*t*
Zhang, 2008 [11]	Rats	11	9/10	1 × 10^6^ + 1 × 10^6^	2 × 10^6^/2 × 10^6^	28 days	84 days	1. Cardiac function and hemodynamics (ejection fraction, FS, LVEDD, LVEDP, +d*p*/d*t*, –d*p*/d*t*)
								2. Capillary density and regional myocardial blood flow
								3. Myocardial fibrosis
								4. Angiogenic growth factor protein and mRNA expression
Yi, 2010 [13]	Rats	6	4/6	0.5 ml + 0.5 ml	1 ml/1 ml	7–10 days	28 days	1. Hemodynamics (LVSP, LVEDP, +d*p*/d*t*, –d*p*/d*t*)
								2. Infarct size
								3. Capillary density
Xiaolin, 2010 [14]	Rats	8	8/8	2 × 10^6^ + 0.5 × 10^7^ × 3	2 × 10^6^	7 days, 14 days	35 days	Cardiac function
Yuhui, 2010 [12]	Rats	10	10/10	5 × 10^6^ + 5 × 10^6^	5 × 10^6^/5 × 10^6^	7 days	28 days	Cardiac function (LVSP, LVEDP, +d*p*/d*t*, –d*p*/d*t*)
Bone								
Zigdon-Giladi, 2015 [17]	Rats	2/8	–	5 × 10^5^ + 5 × 10^5^	–	–	4 weeks and 12 weeks	1. Blood vessel density
								2. Vertical bone height
Zigdon-Giladi, 2014 [18]	Rats	8/6	–	5 × 10^5^ + 5 × 10^5^	–	–	12 weeks	1. Bone volume fraction (BV/TV)
								2. Tissue mineral density (TMD)
Shaolong, 2014 [16]	Rabbits	12	12/12	–	–	14 days	14 days and 28 days	1. Vessel density
Xian, 2013 [8]	Rabbits	6	6/6	–	–	–	3 days, 7 days, and 14 days	1. ALP activity
								2. The OC expression
								3. Cell proliferation activity
Li, 2013 [19]	Dogs	–	–	–	–	–	24 h, 3 days, 7 days, and 14 days	1. ALP activity
								2. Mineralized nodule
								3. Gene expression of key osteogenic markers
								4. Matrigel 2D assay
Seebach, 2012 [20]	Rats	6	6/6	5 × 10^5^	5 × 10^5^/5 × 10^5^	–	1 week	1. Area of neovascularization
								2. VEGF release
Huiping, 2011 [21]	Rats	6	6/6	1 × 10^5^ + 1 × 10^5^	1 × 10^5^/1 × 10^5^	–	–	1. ALP activity
								2. Cell proliferation activity
Fedorovich, 2010 [22]	Goats	–	–	1 × 10^5^ + 1 × 10^5^	–/2 × 10^5^	–	2 weeks, 6 weeks	1. In-vitro analysis of 2D/3D network formation on Matrigel and osteogenic differentiation
								2. In-vivo vascularization and bone formation
Usami, 2009 [23]	Mice	–	–	5 × 10^6^ + 1 × 10^7^	5 × 10^6^/1 × 10^7^	–	7 days, 14 days	1. ALP activity
								2. Soft X-ray
								3. Implant capillary scoring
Cerebral vessels								
Zigen, 2013 [9]	Rats	25	25/25	2 × 10^6^ + 2 × 10^6^	–/2 × 10^6^	1 day	7 days	1. Cerebral infarction volume
								2. BDNF
								3. Neurological score
Yao, 2014 [10]	Rats	16	16/16	2 × 10^6^ + 2 × 10^6^	2 × 10^6^/2 × 10^6^	1 day	28 days	1. Cerebral infarction volume
								2. BDNF
								3. Neurological score
Fenfang, 2014 [15]	Rats	20	20/20	2 × 10^6^ + 2 × 10^6^	2 × 10^6^/2 × 10^6^	26h	7 days	1. The expression of Bcl-2 and Bax
								2. The Neurological Score

*–* not applicable, *ALP* alkaline phosphatase, *BDNF* brain-derived neurotrophic factor, *−d*p*/d*t maximum left ventricular pressure rate of fall, *+d*p*/d*t maximum left ventricular pressure rate of rise, *EPC* endothelial progenitor cell, *FS*, fractional shortening, *LVEDD* left ventricular end-diastolic diametric, *LVEDP* left ventricular end-diastolic pressure, *LVEF* left ventricular ejection fraction, *LVSP* left ventricular systolic pressure, *MSC* mesenchymal stem cell, *OC* osteocalcin, *VEGF* vascular endothelial growth factor

## Discussion

Cell-based therapy has been widely applied in bioengineering, as well as to facilitate tissue repair and regeneration. Neovascularization represents an important process involved in tissue regeneration [17], because a sufficient blood supply is required to ensure the availability of nutrients during tissue repair. The promotion of neovascularization during tissue repair is therefore the focus of intense effort in the field of regenerative medicine.

EPCs which reside in the bone marrow, adult peripheral blood, and human umbilical cord blood differentiate into mature ECs that not only participate in angiogenesis during embryonic development, but also play an important role in microvascular neovascularization and vascular endothelial repair following differentiation [24]. MSCs, which represent important members of the stem cell family, possess the ability to self-renew, differentiate, and participate in angiogenesis [1]. Numerous clinical trials have confirmed that MSCs may be used in the treatment of diseases such as chronic heart infarction, acute myocardial infarction, and hematological malignancies [25]. MSCs and EPCs, which promote vascularization and tissue repair via different pathways, have both been used as seed cells for tissue engineering [26, 27]. Most previous studies have utilized single-cell transplantation, which suffers from several limitations. For several studies, it was reported that strategies involving the combined transplantation of multiple cell types are more effective than single-cell transplantation [23]. This meta-analysis was conducted to access the efficacy of combined cell transplantation therapy in promoting angiogenesis and tissue repair.

Results showed that there was no difference in angiogenesis between the EPC and MSC cotransplantation group and the EPC single-transplantation group; the heterogeneity of the data was 88 %. This heterogeneity resulted from various factors, such as differences in the organization source, injection dose, and measurement time (Table 1). Because of the small amount of available data, we were unable to perform sensitivity analysis. Our findings indicated that the transplantation of EPCs alone achieved the same effect on angiogenesis as combined transplantation; however, MSCs were able to induce differentiation into EPCs under specific conditions [19]. Several studies have reported that inducing the differentiation of EPCs into other cell types is no easier than inducing the differentiation of MSCs [12, 13, 28]. Numerous studies have demonstrated that these cells are capable of differentiating into ECs, thereby contributing to the formation of vascular networks [2, 10–15, 21]. These findings confirmed that EPCs may be utilized to achieve a significant enhancement in angiogenesis and revascularization. EPCs are considered capable of ensuring the availability of sufficient blood supply, thereby ensuring a source of nutrients during tissue repair. However, the present results showed that cell therapies using a combination of MSCs and EPCs may be applied to achieve improved tissue regeneration and repair, relative to the transplantation of either cell type alone. The results revealed that the combined transplantation of MSCs and EPCs achieved an improvement in cardiac function in cardiac diseases, in ALP activity and bone volume in damaged bone tissue, and in cerebral function in cerebrovascular diseases by increasing BDNF and reducing neurologic impairment. However, these results may be affected by the injection dose or organ source, and can also be associated with the injured tissue needing more new vessels to repair, the extent of tissue damage, or the ability of the tissue to regenerate. Our findings confirmed that combination therapy using both MSCs or EPCs is more effective than therapy using MSCs or EPCs alone, under some conditions. MSCs are capable of differentiating into various cell types, thereby providing a source of cells for the repair of damaged tissues; therefore, combination therapy involving the transplantation of these cells achieves improved tissue repair. However, compared with the transplantation of MSCs alone, the effect of combined transplantation on left ventricular function was not clear; the + d*p*/d*t* or –d*p*/d*t* value represented a significant difference, but no difference was observed in LVSP and LVEDP. We speculate that this heterogeneity may be attributed to the varying injection times of EPCs and MSCs reported in the studies: one study reported that EPCs were injected for 3 days (0.5 × 10^7^ EPCs/day) at 7 days post MSC injection [14]. In other studies, both cell types were injected at the same time. These findings suggested that EPCs play a more important role in the early stages of vascularization than MSCs and that MSCs promote EPC proliferation and provide a stable microenvironment for these cells. The observed heterogeneity may also be attributed the reduced capacity of the myocardium to regenerate, or variations in factors such as instrument sensitivity.

To our knowledge, the present meta-analysis is the first to evaluate the effectiveness of combined cell-transplantation therapy. Because this form of cell therapy has not been widely applied in clinical trials to date, most of the included studies reported the efficacy of combination cell therapies from animal studies. Our results are expected to guide preclinical and clinical trials in investigating the efficacy of combination cell therapies. Further investigation is required to determine the optimal cotransplantation dose, therapeutic method, and time of treatment.

### Limitations

Our meta-analysis has several limitations. Since the data were complex and insufficient, we could not assess the heterogeneity or perform sensitivity analyses of studies related to vessel density. However, the main factors that may affect the end evaluation are listed and divided into various groups (Table 1) or presented descriptively.

## Conclusions

Interest in combination cell therapy should increase in the future after better efficacy is achieved. In this study, we collected, read, and analyzed a large amount of articles to investigate the efficacy of combination cell therapy using statistical methods. Unfortunately, we could not perform further research and statistical analysis due to the small number of data, although we could still conclude that EPCs played an important role in vascular regeneration when compared with combined transplantation, which was associated with EPC characteristics such as differentiation into vascular ECs and composing the vascular wall. Combined transplantation could promote robust tissue regeneration. The underlying mechanism of stem/progenitor cell therapy in tissue repair is presently unclear. MSCs/EPCs can secrete several nutritional factors to promote the repair of tissue functions after entering into tissues, so as to continuously improve tissue functions and promote the regeneration of surrounding nerves. In addition, MSCs/EPCs may activate or promote the homing of other cells to damaged tissues during the regeneration of tissue cells [29, 30]. Recent research has only superficially investigated their regenerative abilities in tissues, and detailed analysis of the effects of the number of injected cells, injection mode, and other parameters has not been performed. Further research into these issues needs to be conducted before cell-based therapy can be safely and effectively applied in a clinical setting.
